# Supplementary material for: Rapid turnover of life-cycle-related genes in the brown algae
Source: Genome Biol. 2019 Feb 14;20:35. doi: 10.1186/s13059-019-1630-6 (PMC6374913; doi:10.1186/s13059-019-1630-6)
Supplement: Supplementary file 3 — Protein alignments, corresponding Gblocks html file and CDS sequences for the lineage conserved gametophyte-biased genes. (ZIP 2109 kb) [file 13059_2019_1630_MOESM3_ESM.zip › Con_GA_alignments/Ec-26_006030.aa_ali.fasta-gb.htm]

Ec-26\_006030.aa.fasta.trans.aa\_ali.fasta


## Gblocks 0.91b Results

Processed file: **Ec-26\_006030.aa.fasta.trans.aa\_ali.fasta**  
Number of sequences: **2**  
Alignment assumed to be: **Protein**  
New number of positions: **1031** (selected positions are underlined in blue)

```
                         10        20        30        40        50        60
                 =========+=========+=========+=========+=========+=========+
Ec-26_006030     MGLSFRASRVLERLHNAIQVRGQHGLDEFRARLDEQRLHDKREATETEFIAAAGGDDGVL
MSTRG.45323.1    ------------------------------------------------------------
                                                                             


                         70        80        90       100       110       120
                 =========+=========+=========+=========+=========+=========+
Ec-26_006030     SGDEAAILFRELGGKDKSIPFAEIIACYRANNHPYVRRGEKASAAVQQDFLHTLSNITST
MSTRG.45323.1    -----------------------IRVWYHAPKHPDVRRGLRTSTDVRQDFLQTLFDVMSV
                                        #####################################


                        130       140       150       160       170       180
                 =========+=========+=========+=========+=========+=========+
Ec-26_006030     DLHNSHGTMVSFPAFLDFYCSLTAFTSDTDFRDAIVAVWVPPVVLDNDHVLHTLGAAGGL
MSTRG.45323.1    DAHSNGQEMVSFPSFLDFYCSLTAFTSDDDFRDAILAVWVPPVACGNEDIPHTLGAAGGL
                 ############################################################


                        190       200       210       220       230       240
                 =========+=========+=========+=========+=========+=========+
Ec-26_006030     VAKLRSQILTNGLPGVAAFCRGVRKAARGSDGGGKEITQEAFRAAASSAGASLTASEMSQ
MSTRG.45323.1    VANLRSQILAKGLAGVVAFCRGVRKLAVRGEGDSQEITQEAFQAAASFAGAKLTASEMSQ
                 ############################################################


                        250       260       270       280       290       300
                 =========+=========+=========+=========+=========+=========+
Ec-26_006030     AFRHLDRRGVGKIYVAGALRAVRGEISKRREHVIDEVFDSLLFRLGGDSDAGDGQHSHTL
MSTRG.45323.1    AFRHLDRRGVGHVSVVDALRAVRGEVNTRRADVIDDTFDALLFRSDHES-GRSGQDSQTL
                 #################################################    #######


                        310       320       330       340       350       360
                 =========+=========+=========+=========+=========+=========+
Ec-26_006030     EPVAVAKLYRAEAHPDVVSGVRTQAEVLSEFLEAFEGGGEIDGRVTQGEWREHHLQVSAT
MSTRG.45323.1    EPSAISKLFRAEEHPDVVSGARSQGEVSSEFLESFDGGGEIGGRVSRAEWRDHHSHVSAL
                 ############################################################


                        370       380       390       400       410       420
                 =========+=========+=========+=========+=========+=========+
Ec-26_006030     MADDDLFCEHMKCVWGYGNKEKATLEASHVSNHDLTGSTTTNNYNTAIRGPNGAHGSVEQ
MSTRG.45323.1    IPDDDAFYEHVKRVWGCDDRRSSVRDSSNSNNKGLQRNTLDDVQNSPTRDSTSAVGDAEE
                 ################           #################################


                        430       440       450       460       470       480
                 =========+=========+=========+=========+=========+=========+
Ec-26_006030     GDGNSVSSKSN--GDGRQRGGEAGANSPFAAREAWAPSPPAASNAERGPILHQGSARDAA
MSTRG.45323.1    PGGNFTLANNNSACQGRHEHRAGTVAAPPVSREAWALSSRPASTTGENPTINGDIARAAT
                 ###########                ################################ 


                        490       500       510       520       530       540
                 =========+=========+=========+=========+=========+=========+
Ec-26_006030     TAAR-LSPGVLGLLARARGSLASGGMRAAFQLLKGFREEDQGDDGKVTLSGFKKAIGGAA
MSTRG.45323.1    STRSNIPPGILGILRRVRSSLASGGMRAAFALLKGFREGDQEGNGKVKLAAFKKAVSGAA
                        #####################################################


                        550       560       570       580       590       600
                 =========+=========+=========+=========+=========+=========+
Ec-26_006030     LGLGLKEAEMRIIFQHFDKEGDGEVPYELFLRHIRTTLPDGRLALAHEVFDRMDADGDGL
MSTRG.45323.1    R--GLKEAEMRIIFQHFDEGGDGEVAYEQFLRHVRETLPEGRLALANKVFDRMDADGNGL
                    #########################################################


                        610       620       630       640       650       660
                 =========+=========+=========+=========+=========+=========+
Ec-26_006030     VFVDDVARTFRAGTHPEVVEGSRTEEDVRREFLESFSVTDGSHKWCTRDDLIWWMELLSS
MSTRG.45323.1    VSVDDVALNFKASAHPEVVEGSREEEDVRREFLESFPVANGSHKWCTRDDVIWWMELLSS
                 ############################################################


                        670       680       690       700       710       720
                 =========+=========+=========+=========+=========+=========+
Ec-26_006030     SLPNATPAADDDLQFERVARACWGIPVNAPGMPGSQQDGAFGEAPQYVSVVVTHADGSIT
MSTRG.45323.1    SLP-ITPSG--DVSFESLVRACWGIPADPVGGSGSQANNGKGDD-TSISVVVTHADGSMS
                 ###        ###############################      ############


                        730       740       750       760       770       780
                 =========+=========+=========+=========+=========+=========+
Ec-26_006030     TERVSRQEVEVHP-DGSG----TLLISEVQAENIRRQLSRRGVHAVHVQLLNEQK-GTVN
MSTRG.45323.1    VERVPWHAAGVQSGSGDGTDGDDLLVSQMQAEEIRRYLFLRGVHAVHVHLLKKPRGGTAH
                 ###########            ############################     ####


                        790       800       810       820       830       840
                 =========+=========+=========+=========+=========+=========+
Ec-26_006030     ASPEAAGSSQETADVKHAGSTILEG-SSCSRPHGVLQLSADEVRTARLGPSGHGQQSRGA
MSTRG.45323.1    ATQSGTGNVAKGGLLVNNTGALLDGQSPRTLLHDGRRPSFDEVRVARFGPSGDGKGNEGG
                 #######                   ##################################


                        850       860       870       880       890       900
                 =========+=========+=========+=========+=========+=========+
Ec-26_006030     DCFGEGEANLGFDHGMLSVLHMARRQLALRGPVGVFALSKRLRSAAYFRSKAAPSSHPAF
MSTRG.45323.1    GCFEEDDPGLSFDHGLRSVLQVARKRVALRGTAGFFSLSKRLRSAGYFRAGAASSSQRAT
                 ############################################################


                        910       920       930       940       950       960
                 =========+=========+=========+=========+=========+=========+
Ec-26_006030     VAAAAAAAAASMANSSSGVAATDR-NTGPFLSLGAFKDALSEVNCGLSGKDLTNIFTHLD
MSTRG.45323.1    VAAAAAAAAAASSGYVGIYSGADTPNCGPFVSLEGFREALSEVGCGLDGKDLTTIFAHLD
                 ##########               ###################################


                        970       980       990      1000      1010      1020
                 =========+=========+=========+=========+=========+=========+
Ec-26_006030     DAGRGEVLVDVALEAFRGRLTGVRLALVRAAFTRLRGSGDAPPDAVAAVNVAFQFNPRGH
MSTRG.45323.1    NRSVGEIPVEMALEAIRGRLTGMRLSLVRAAFTRLQSSGDGPPDAAAAVNVAFQYDPAGH
                 ############################################################


                       1030      1040      1050      1060      1070      1080
                 =========+=========+=========+=========+=========+=========+
Ec-26_006030     PDVIAGRRSPEDVRKDFMDALTTTKGFVTRKDFESYYTDVSAATPSDAYFSLQIQACWGL
MSTRG.45323.1    PEVIAGRRAPEDVRKEFMDALTTTEGFVTRKDFESYYSDVSAATPSDGYFALLVQACWGL
                 ############################################################


                       1090      1100      1110      1120      1130      1140
                 =========+=========+=========+=========+=========+=========+
Ec-26_006030     GRATIDSTAAFADANTAPHSGTGAPAGEHTAGLGRDYDPAKSRRTYSKARTSAAPGVLPG
MSTRG.45323.1    G----QAAAASAASNTVAIPGT-MAAGETNVRLNGDHDPAITRRTYSKARTSAPLGVLPG
                 #       ##############   ###################################


                       1150      1160      1170      1180      1190      1200
                 =========+=========+=========+=========+=========+=========+
Ec-26_006030     TAERG-----DKPAAVAVRWGDQSGMRTPTAGVAAILRKLGKEVQGHGAAWGLAGLRRAL
MSTRG.45323.1    TAARSNIAASAVAARAVARRVPRGGVRTPTAGVAAILRKLGTEVQGNGAAWGLAGLRRAL
                 ####         ###############################################


                       1210      1220      1230      1240      1250      1260
                 =========+=========+=========+=========+=========+=========+
Ec-26_006030     FNADGNKDGFLSFGDLKAALSSSRAGLTIPEVRALFTHLETEGGPRRAGGGGGDGSGVVP
MSTRG.45323.1    FDADRDKDGLLSFGDLKAALSSSRAGLTTPEVRALFAHLE--------------------
                 ########################################                    


                       1270      1280      1290      1300      1310      1320
                 =========+=========+=========+=========+=========+=========+
Ec-26_006030     WQALLDATRPPLSGERLGLVRLAFGKMDKEGRGHVSPETVTQRFVGSRHPAVLRGHATAE
MSTRG.45323.1    ------------------------------------------------------------
                                                                             


                       1330      1340      1350      1360      1370      1380
                 =========+=========+=========+=========+=========+=========+
Ec-26_006030     EVRQELLDTFFSAGASETTAREEQVSEKDFIEHHANLSFGIKRDEDFRELVINCWGLDGG
MSTRG.45323.1    ------------------------------------------------------------
                                                                             


                       1390      1400      1410      1420      1430      1440
                 =========+=========+=========+=========+=========+=========+
Ec-26_006030     AVGDGDVCMPADVADMLRRVNAPRRKFLVSYADGSQRVEELPFSSKGSEQREAEGSLTAA
MSTRG.45323.1    ------------------------------------------------------------
                                                                             


                       1450      1460      1470      1480      1490      1500
                 =========+=========+=========+=========+=========+=========+
Ec-26_006030     EERVLKRELLKRDDIHATQVTLLPRTYSEEGGPHSALESSLASAQENPAAVDHRPAWTWA
MSTRG.45323.1    ------------------------------------------------------------
                                                                             


                       1510      1520      1530      1540      1550      1560
                 =========+=========+=========+=========+=========+=========+
Ec-26_006030     PMNLSEVQDAAAAAANAAAPESSVHEETGNQHDQSGLHGSLVSSDGIGKENDKRPAVGDR
MSTRG.45323.1    ------------------------------------------------------------
                                                                             


                       1570      1580      1590      1600      1610      1620
                 =========+=========+=========+=========+=========+=========+
Ec-26_006030     NEFDATEVVEVDWEGEEGDGGGAGGRARQRMRAGGRRIQAGEGREADLSMIYLLSKLRSE
MSTRG.45323.1    ------------------------------------------------------------
                                                                             


                       1630      1640      1650      1660      1670      1680
                 =========+=========+=========+=========+=========+=========+
Ec-26_006030     LHASGISGMANLIRRLREAAVLLEIKGKESCYGVGEVDVRRALREANLPLTESDIRRIFS
MSTRG.45323.1    ------------------------------------------------------------
                                                                             


                       1690      1700      1710      1720      1730      1740
                 =========+=========+=========+=========+=========+=========+
Ec-26_006030     HFEMGQSGVMNLGALMDRLSGCMGNGRVVLVDEAFDRLDTSGQGHLTLDKVIDSFDATKH
MSTRG.45323.1    ------------------------------------------------------------
                                                                             


                       1750      1760      1770      1780      1790      1800
                 =========+=========+=========+=========+=========+=========+
Ec-26_006030     PAVVSGRQTPDSVFRDFVDGFIAGFPPDKRDGARVTKEDFRRHYDVISATIPDDGYFELL
MSTRG.45323.1    ------------------------------------------------------------
                                                                             


                       1810      1820      1830      1840      1850
                 =========+=========+=========+=========+=========+======
Ec-26_006030     LRSCWRMRGPSGVAGVANTQDGSFVPPGGDTRGDDGSNERNPPSQARDNGVYPIGN
MSTRG.45323.1    --------------------------------------------------------
```

```
Parameters used
Minimum Number Of Sequences For A Conserved Position: 2
Minimum Number Of Sequences For A Flanking Position: 2
Maximum Number Of Contiguous Nonconserved Positions: 8
Minimum Length Of A Block: 10
Allowed Gap Positions: None
Use Similarity Matrices: Yes
```

```
Flank positions of the 15 selected block(s)
Flanks: [84  289]  [294  376]  [388  431]  [448  479]  [488  540]  [544  663]  [672  702]  [709  731]  [744  771]  [777  787]  [807  910]  [926  1081]  [1089  1102]  [1106  1144]  [1154  1240]  

New number of positions in Ec-26_006030.aa.fasta.trans.aa_ali.fasta-gb:  1031  (55% of the original 1856 positions)
```
